# A comparative study using Xpert MTB/RIF and culture methods evaluates MassARRAY technology for rapid detection of *Mycobacterium tuberculosis* and drug resistance

**DOI:** 10.3389/fcimb.2025.1539240

**Published:** 2025-07-09

**Authors:** Sufang Wu, Lulu Ge, Shulan Zheng, Xiaocui Ma, Ruixia Liang, Baolong Zhang

**Affiliations:** ^1^ Department of Tuberculosis, Henan Provincial Chest Hospital, Zhengzhou University, Zhengzhou, Henan, China; ^2^ Tuberculosis Clinical Research Center of Henan Province, Zhengzhou, Henan, China; ^3^ Children`s Hospital Affiliated to Zhengzhou University, Henan Key Laboratory of Pediatric Genetics and Metabolic Diseases, Zhengzhou, Henan, China; ^4^ Institute for Systems Biology, School of Life Sciences, Jianghan University, Wuhan, Hubei, China

**Keywords:** tuberculosis, *Mycobacterium tuberculosis*, MassARRAY, drug resistance, molecular diagnosis

## Abstract

Tuberculosis (TB) remains a major global health threat, with the urgent need for rapid and accurate diagnostic methods to improve control and treatment outcomes. This study evaluates the performance of MassARRAY technology for detecting *Mycobacterium tuberculosis* (MTB) and identifying drug resistance, compared to traditional culture methods and Xpert MTB/RIF. From July 2021 to February 2024, bronchoalveolar lavage fluid (BALF) samples from 289 suspected pulmonary tuberculosis patients at Henan Provincial Chest Hospital, China, were tested using MassARRAY, Xpert MTB/RIF, and conventional culturing techniques. The performance of each method was assessed for MTB detection, and the ability of MassARRAY to identify drug resistance was compared with standard drug susceptibility testing (DST). MassARRAY demonstrated a sensitivity of 96.5% and a specificity of 34.6% for MTB detection, outperforming the Xpert MTB/RIF assay in sensitivity (94.7%) but showing lower specificity. In detecting rifampicin resistance, MassARRAY achieved concordance rates of 83.93% with Xpert MTB/RIF and 72.73% with DST. Furthermore, MassARRAY successfully identified key genetic mutations associated with drug resistance, such as *rpoB 531* for rifampicin and *katG 315* for isoniazid. MassARRAY demonstrated high concordance with DST for several drugs, including isoniazid, kanamycin, and streptomycin, but exhibited limitations in detecting resistance to pyrazinamide, clofazimine, cycloserine, and linezolid. Overall, MassARRAY provides a rapid, cost-effective, and high-throughput diagnostic platform for MTB and drug resistance, particularly for first-line anti-tuberculosis drugs. While limitations in specificity and resistance detection for certain second-line drugs exist, its ability to rapidly provide comprehensive resistance profiles makes it a valuable tool for TB management.

## 1 Introduction

Tuberculosis (TB) is a chronic infectious disease caused by *M. tuberculosis* which remains one of the leading causes of morbidity and mortality, representing a major threat to global health. Tuberculosis was the second deadliest infectious disease in worldwide 2022, following COVID-19. According to World Health Organization report around ten million individuals get affected by tuberculosis every year and nearly one and half million of them resulting in fatalities (WHO, 2023a). In addition to human-to-human transmission, tuberculosis can also be zoonotic in nature, primarily caused by *Mycobacterium bovis*, particularly from dairy animals (Aljohani, 2023). Treatment for TB typically involves the use of multiple antibiotics combined over several months or years. The recent appearance of multidrug-resistant TB (MDR-TB) and extensively drug-resistant TB (XDR-TB), has complicated treatment, diminished cure rates, increased cost of management significantly (Dheda et al., 2017). Hence, the early and accurate diagnosis of *M. tuberculosis* and drug resistance phenotypes is important for both epidemic control and improving cure rates.

The diagnosis of *M. tuberculosis* is currently based on a combination of microbiological methods, molecular biological tests and imaging examination. One of these is the gold standard, traditional culture approach, though reliable, is time-consuming, often delaying treatment initiation. Smear microscopy is the other, although it provides data more quickly, it is less sensitive (Dong et al., 2022). With results in as little as two hours, Xpert MTB/RIF, the most used molecular diagnostic technique, is a fluorescent semi-nested real-time PCR for detecting *M. tuberculosis* and rifampicin resistance. However, this method was limited to detecting rifampicin resistance and does not provide comprehensive drug resistance profiles (Irfan and Bisht, 2022). While there are many uses for digital PCR technology in the detection of *M. tuberculosis*, issues related to its high cost, complexity and extended detection periods remain unresolved (Vogelstein and Kinzler, 1999; Devonshire et al., 2015). To address these issues, MassARRAY technology employs matrix-assisted laser desorption/ionization time-of-flight mass spectrometry (MALDI-TOF MS) to offer a rapid means of pathogen identification and the detection of drug-resistant gene. When compared to traditional testing techniques, MassARRAY offers several significant advantages: higher output volume, enhanced sensitivity, faster processing speed, and greater cost-effectiveness (Yoon and Jeong, 2021; Yang et al., 2023).

Several studies have confirmed the effectiveness of MassARRAY technology in the diagnosis of *M. tuberculosis*. For example, Wang et al. investigated the application of MALDI-TOF MS in the rapid diagnosis of pulmonary tuberculosis and detection of drug resistance. Their results showed that the sensitivity and specificity of this technique for MTB detection were 92.2% and 74.1%, respectively, exceeding traditional culture methods (Wang et al., 2023). Li et al. further optimized the analysis process of MALDI-TOF MS, validating its high sensitivity (96.91%) and specificity (100%) in the identification of *M. tuberculosis* (Li et al., 2022). In addition to diagnosing *M. tuberculosis*, MALDI-TOF MS has also been applied in detecting drug resistance. Wu et al. evaluated its performance in predicting *M. tuberculosis* drug resistance, showing that its sensitivity for detecting rifampicin and isoniazid resistance was 92.2% and 90.9%, respectively, showing strong consistency with phenotypic drug susceptibility testing (DST) (Wu et al., 2022). Shi and colleagues investigated the use of MassARRAY in retreatment patients, discovering that MALDI-TOF MS was highly consistent with the BACTEC 960 liquid culture method in detecting resistance to various anti-tuberculosis drugs (Shi et al., 2022). Furthermore, MALDI-TOF MS can also be applied to the detection of other pathogens. Yao et al. evaluated its use in detecting non-tuberculous mycobacteria (NTM), showing that the sensitivity and specificity for NTM detection were 77.8% and 92.5%, respectively (Yao et al., 2023). Although previous research had shown that MassARRAY technology has considerable advantages in the detection of *M. tuberculosis* and their drug resistance, there are several limitations to these investigations. Some studies were restricted by small samples size, reducing the generalizability of results. Some were focused on resistance testing for a particular treatment and did not fully cover the spectrum of anti-tuberculosis drugs, and some procedures had specific flaws that could contribute to false positive results (Wang et al., 2023; Gao et al., 2024; Liang et al., 2024).

The purpose of this study is to evaluate the potential of MassARRAY technology in drug resistance analysis and rapid tuberculosis diagnosis. It will also compare it to conventional cultivation methods and the commonly used Xpert MTB/RIF analysis. Initially, the study will evaluate MassARRAY’s sensitivity and specificity in detecting *M. tuberculosis* in the clinical sample. The study will then evaluate MassARRAY’s efficacy in detecting drug resistance in *M. tuberculosis*, including resistance to rifampicin, isoniazid, ethambutol, and other relevant medications. By fulfilling these goals, the research hopes to offer new technical assistance for the improvement of TB control tactics as well as scientific proof for the rapid diagnosis of tuberculosis.

## 2 Methods

### 2.1 Ethical declaration

This study was approved by the Ethics Committee of Henan Provincial Chest Hospital (Ethics Review Approval Number: 2024-05-11), and all participants signed an informed consent form.

### 2.2 Participant recruitment

Samples of bronchoalveolar lavage fluid (BALF) from 289 patients suspected of pulmonary tuberculosis, who visited Henan Provincial Chest Hospital from July 2021 to February 2024, were chosen for the study (**Supplementary Table S1**). Of the 289 patients included, 54.3% were male, and 45.7% were female, with a mean age of 46.7 years (range: 18–76 years). Inclusion criteria included: (1) Aged 18 years and older; (2) Clinical suspicion of tuberculosis, diagnosis according to WHO guidelines (WHO, 2020); (3) Informed consent obtained. Exclusion criteria: (1) Below 18 years of age; (2) Serious impairment of cardiac, hepatic, or renal function; (3) Pregnant or lactating women; (4) Refusal to participate in the study.

### 2.3 Culture of Mycobacterium tuberculosis

The processed bronchoalveolar lavage fluid (BALF) samples were inoculated into the MGIT 960 culture system (BD Diagnostic Systems, NJ, USA) and incubated in a constant temperature incubator at 37°C (Hanna et al., 1999). All isolated *M. tuberculosis* strains were confirmed through growth tests on para-nitrobenzoic acid (PNB) media and the MBP 64 antigen detection kit (Abe et al., 1999).

### 2.4 Xpert MTB/RIF assay

Processed bronchoalveolar lavage fluid (BALF) specimens were analyzed with the Xpert MTB/RIF testing system (Cepheid Inc., Sunnyvale, CA, USA) (Mechal et al., 2019). In brief, the centrifuged BALF samples were thoroughly mixed with 2mL of sample reagent and incubated at room temperature for 15 minutes. Subsequently, the mixture was transferred to an Xpert MTB/RIF cartridge for automated analysis in the GeneXpert system. This system automates the extraction, amplification, and detection of nucleic acids, providing results within 2 hours.

### 2.5 MassARRAY testing

The identification of *M. tuberculosis* was conducted using MALDI-TOF-MS technology in this study (Li et al., 2022). In brief, sample DNA was first extracted using the MagPure DNA Kit, followed by multiplex PCR amplification for the detection of *M. tuberculosis* and its drug-resistant genes. Specific target genes and sites are detailed in **Table 1**. The PCR reaction system included 10×PCR buffer, MgCl_2_, UNG, dUTP/dNTP Mix, PCR enzyme, and DNA template. The reaction conditions included pre-denaturation at 25°C for 5 minutes, initial denaturation at 95°C for 2 minutes, followed by 45 cycles (denaturation at 95°C for 30 seconds, annealing at 56°C for 30 seconds, extension at 72°C for 60 seconds), and a final extension at 72°C for 5 minutes. The amplification products were processed with SAP mix (containing SAP buffer and SAP enzyme) before the extension reaction, which involved iPLEX buffer plus, iPLEX termination mix, iPLEX pro enzyme, and iPLEX plus extend primer mix, with reaction conditions including denaturation at 95°C for 30 seconds, followed by 40 cycles (denaturation at 94°C for 5 seconds, annealing at 52°C for 5 seconds, extension at 80°C for 5 seconds), and a final 3-minute extension at 72°C. The reaction products were desalted automatically using the MASSARRAY^®^ instrument, then subjected to mass spectrometry analysis with the MassARRAY^®^ Typer, followed by analysis through a bioinformatics workflow based on Python 3. Sample positivity was assessed by calculating the extension rate of measurement sites, with values exceeding the set threshold considered positive.

**Table 1:** List of target drug-resistant genes and loci of the MassARRAY platform.

Drug	Target gene	Included locus
Rifampin (RIF)	*rpoB*	511, 513, 516, 522, 526, 531, 533
Isoniazid (INH)	*inhA*	-15
	*katG*	315, 316
Pyrazinamide (PZA)	*pncA*	57
Ethambutol (EMB)	*embB*	306, 406
Fluoroquinolones (FQs)	*gyrA*	88, 90, 91, 94
	*gyrB*	538, 543, 551
Streptomycin (Sm)	*rpsL*	43, 88
Amikacin (AK)	*rrs*	1,401, 1,484
Kanamycin (Kan)	*eis*	-14
	*rrs*	1,401, 1,402, 1,408
Capreomycin (Cm)	*rrs*	1,401, 1,402, 1,408
Ethionamide (ETO)/protionamide (PTO)	*inhA*	-15
Cycloserine (Cs)	*alr*	113, 261
	*ald*	32
p-aminosalicylic acid (PAS)	*folC*	43
	*thyA*	202, 75
Bedaquiline (BDQ)	*Rv0678*	193, 466
Clofazimine (CFZ)	*Rv0678*	193, 466
Linezolid (LZD)	*rplC*	450

### 2.6 Drug susceptibility testing

Drug susceptibility testing (DST) was conducted using the MicroDST™ assay kit (Sun et al., 2023). Critical concentrations for rifampin, isoniazid, ethambutol, amikacin, capreomycin, para-aminosalicylic acid, moxifloxacin, streptomycin, ofloxacin, and prothionamide were set at 1.0 μg/mL, 0.2 μg/mL, 5.0 μg/mL, 1.0 μg/mL, 2.0 mg/mL, 2.0 mg/mL, 0.5 μg/mL, 2.0 μg/mL, 2.0 μg/mL, and 2.5 mg/mL, respectively. Phenotypic susceptibility testing for pyrazinamide was conducted on Middlebrook 7H10 agar, with a critical concentration established at 100.0 μg/mL. After extraction and adjustment to specified concentrations, strains cultured in liquid media were inoculated onto both drug-containing and drug-free control media and incubated at 36°C ± 1°C for 14 days. Strain sensitivity to each drug was assessed by comparing colony growth on drug-containing media and drug-free control media (Baso, Zhuhai, China).

### 2.7 Statistical analysis

Sensitivity was calculated as the proportion of true positive cases among all cases with the disease (true positives/[true positives + false negatives]). Specificity was calculated as the proportion of true negative cases among all cases without the disease (true negatives/[true negatives + false positives]). Positive Predictive Value (PPV) reflects the proportion of true positive cases among all positive test results (true positives/[true positives + false positives]). Negative Predictive Value (NPV) reflects the proportion of true negative cases among all negative test results (true negatives/[true negatives + false negatives]). Concordance between different detection assays was calculated as the number of overlapping positive results between two methods, divided by the total number of positive cases detected by the reference method.

## 3 Results

### 3.1 Comparative analysis of *M. tuberculosis* identification methods

**Figure 1:**
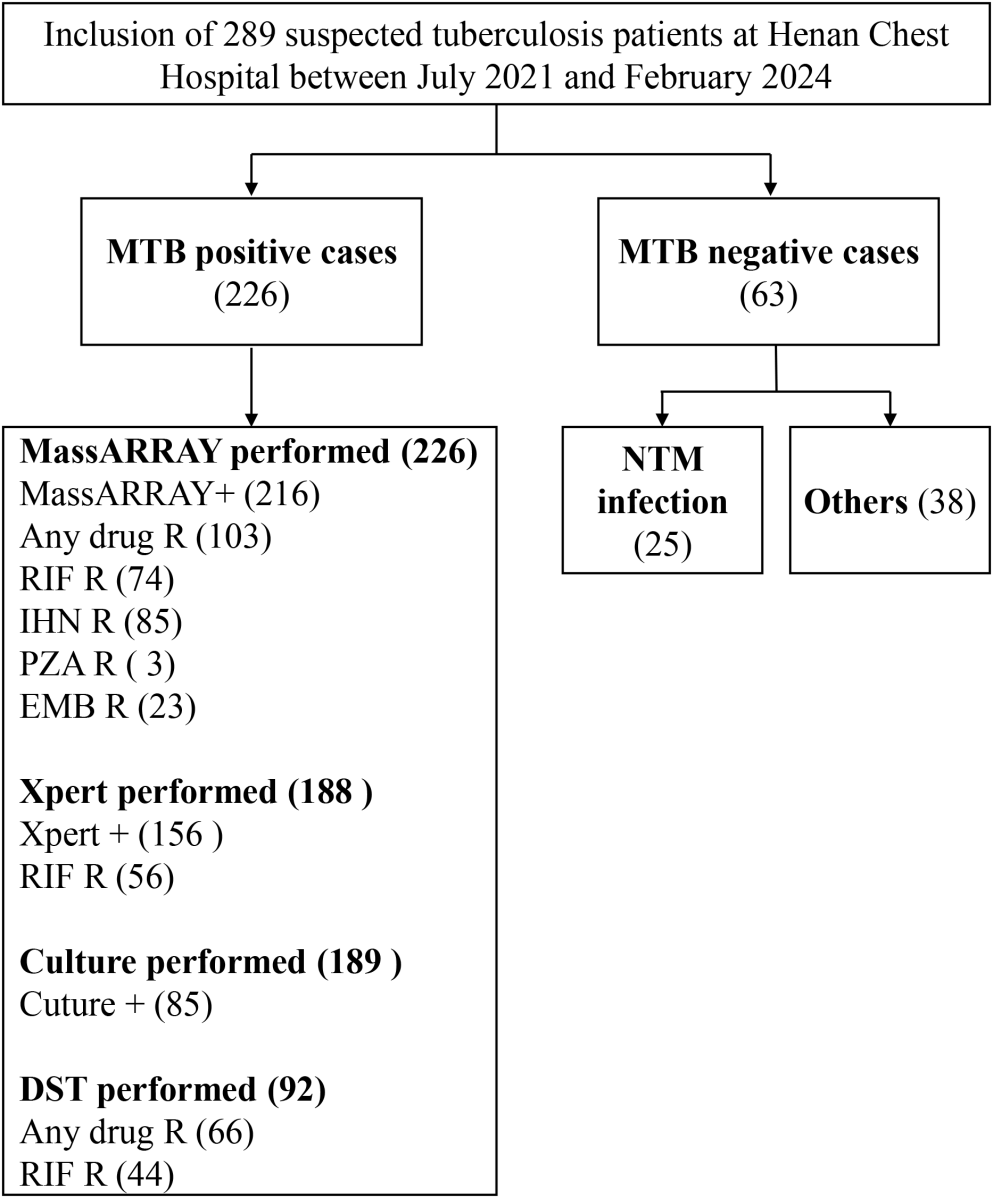
Workflow overview of *M. tuberculosis* identification and drug resistance analysis.

**Figure 2:**
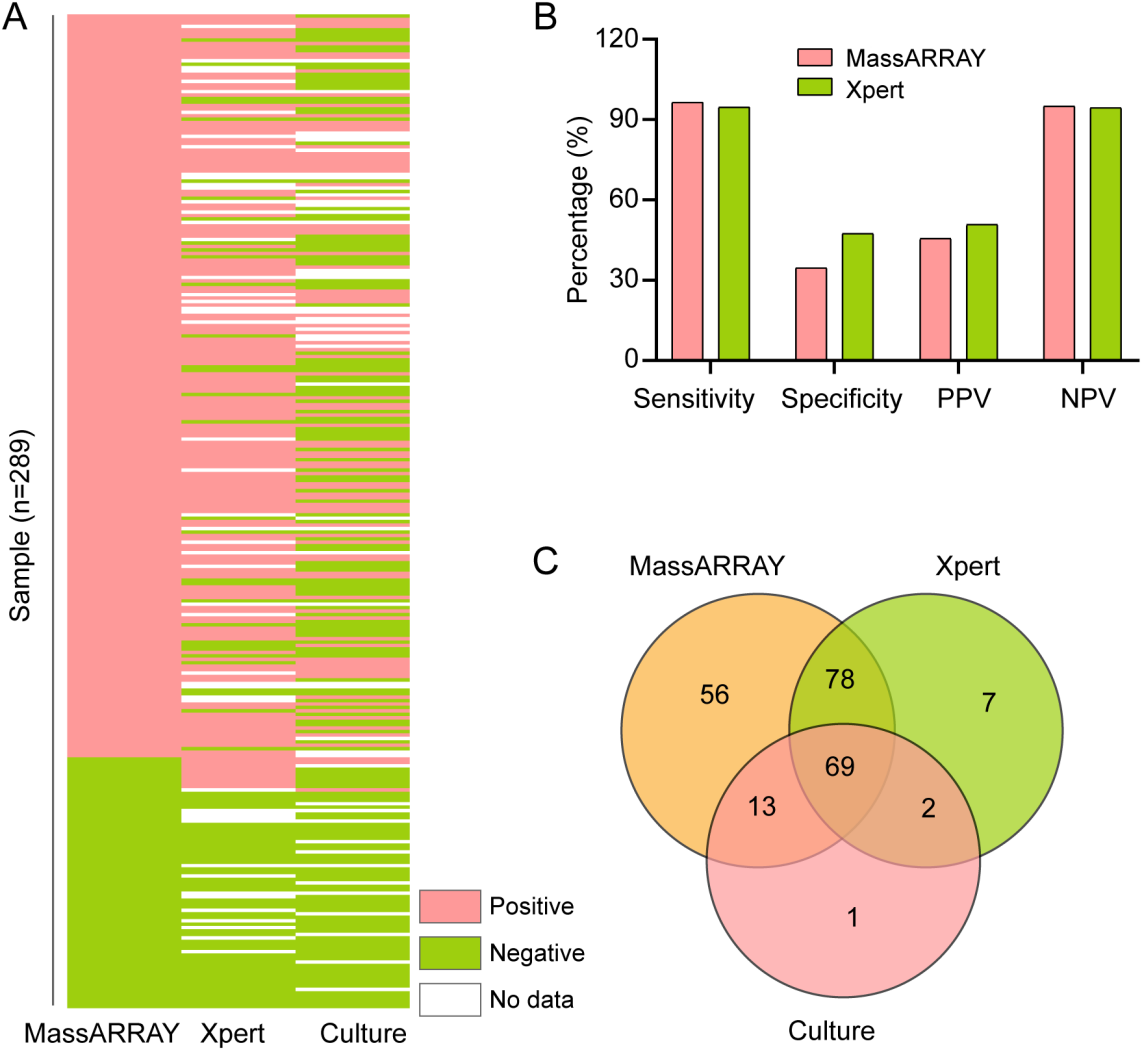
Comparative analysis of *M. tuberculosis* identification methods. **(A)** Comparative performance of MassARRAY, Xpert, and culture methods for *M. tuberculosis* identification. **(B)** Comparison of Sensitivity, Specificity, PPV, and NPV among MassARRAY, Xpert, and culture methods. **(C)** Venn diagram illustrating the overlap of *M. tuberculosis*-positive samples identified by MassARRAY, Xpert, and culture methods.

To evaluate the effectiveness of the MassARRAY method in identifying *M. tuberculosis*, we compared it with the Xpert and culture methods. The study included 289 tuberculosis suspects from Henan Provincial Chest Hospital (**Figure 1**). We used the culture results as the gold standard for *M.tuberculosis* positivity. A total of 240 samples were processed by culture testing, of which 85 were positive for *M. tuberculosis* (**Figure 2A**). Compared to this gold standard, the sensitivity, specificity, positive predictive value (PPV), and negative predictive value (NPV) of the MassARRAY method were 96.5% (95% CI: 90.1–98.8), 34.6% (95% CI: 27.0–43.0), 45.5% (95% CI: 41.1–56.0), and 95% (95% CI: 83.5–97.9), respectively. Meanwhile, the sensitivity, specificity, PPV, and NPV of the Xpert method were 94.7% (95% CI: 87.1–97.9), 47.4% (95% CI: 38.6–56.4), 50.7% (95% CI: 45.3–62.1), and 94.4% (95% CI: 83.8–97.3), respectively (**Figure 2B**). Furthermore, the Venn diagram demonstrated the overlap of positive samples identified by the three methods, of which 69 cases were detected as positive by all methods (**Figure 2C**; **Supplementary Table S2**). Overall, MassARRAY demonstrates high sensitivity and strong diagnostic performance in the identification of *M. tuberculosis*.

### 3.2 Comparative analysis of Rifampicin resistance detection by the MassARRAY

**Figure 3:**
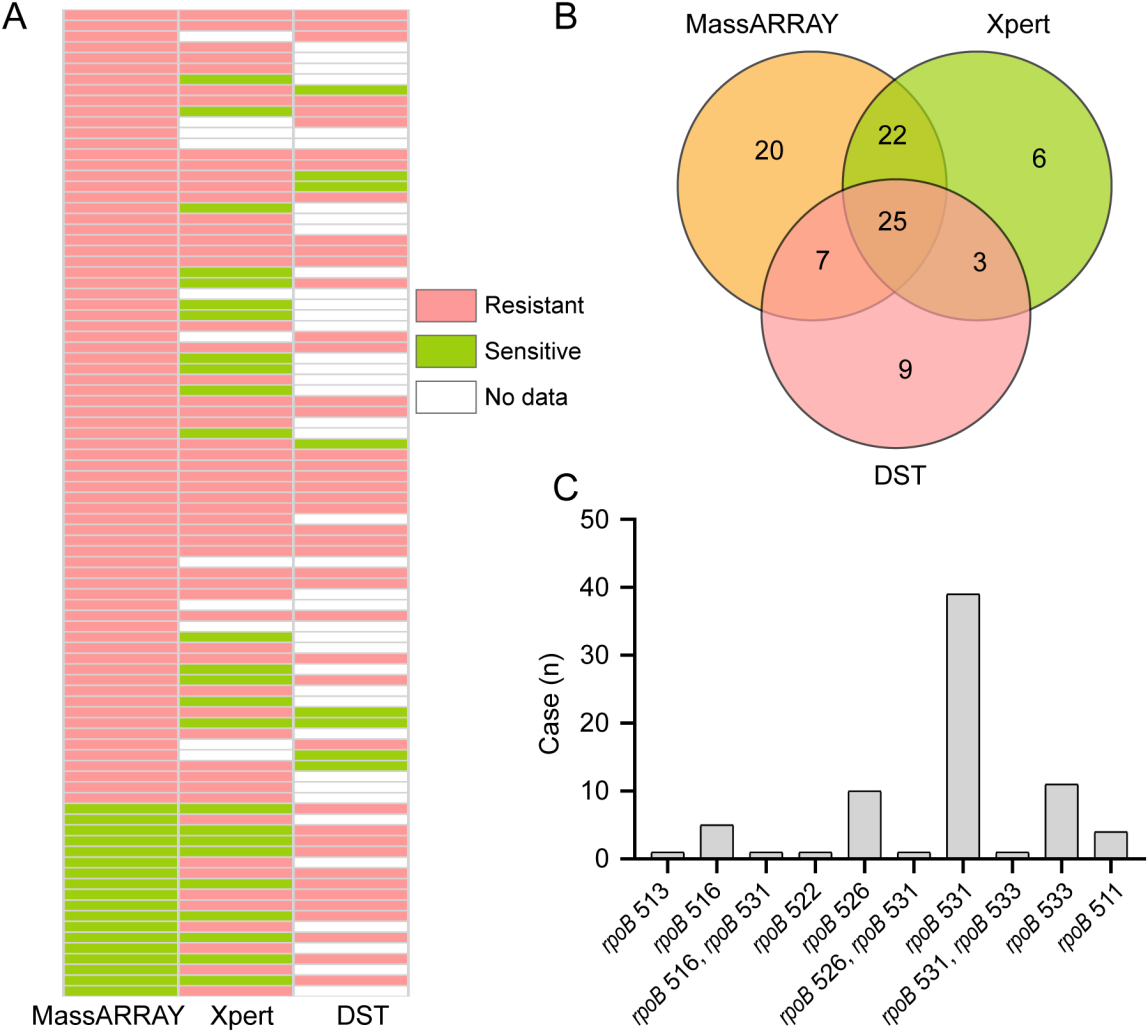
Comparative analysis of Rifampicin resistance detection methods. **(A)** Comparative performance of MassARRAY, Xpert, and Drug Susceptibility Testing (DST) methods for rifampicin resistance detection. **(B)** Venn diagram illustrating the overlap of rifampicin-resistant samples identified by MassARRAY, Xpert, and DST methods. **(C)** Distribution of rifampicin resistance mutation sites identified by the MassARRAY.

In order to explore the effect of the MassARRAY method on the detection of *M. tuberculosis* resistance to rifampicin, we compared it with the Xpert method and the drug sensitivity test (DST). The diagnostic criterion for rifampicin resistance was set as resistance detected by any one of the methods (MassARRAY, Xpert, or DST). Under this criterion, a total of 92 rifampicin resistance cases were identified, with MassARRAY, Xpert, and DST detecting 74, 56, and 44 cases, respectively (**Figure 3A**). In comparison with Xpert, the concordance of MassARRAY in detecting rifampicin resistance was 83.93% (47/56), and with DST, it was 72.73% (32/44). The Venn diagram shows that 25 cases of resistance were identified by all three methods (**Figure 3B**). Additionally, MassARRAY can also analyze the location of mutations in resistant genes. The highest mutation rate was observed at *rpoB* 531 (52.7%), followed by *rpoB* 533 (14.9%) and *rpoB* 526 (13.5%). Three cases were also found with mutations at two sites simultaneously (**Figure 3C**). Overall, MassARRAY has not only high sensitivity but also good specificity in the detection of rifampicin resistance in *M. tuberculosis*.

### 3.3 Effectiveness of MassARRAY method in detecting drug resistance of *M. tuberculosis*

**Figure 4:**
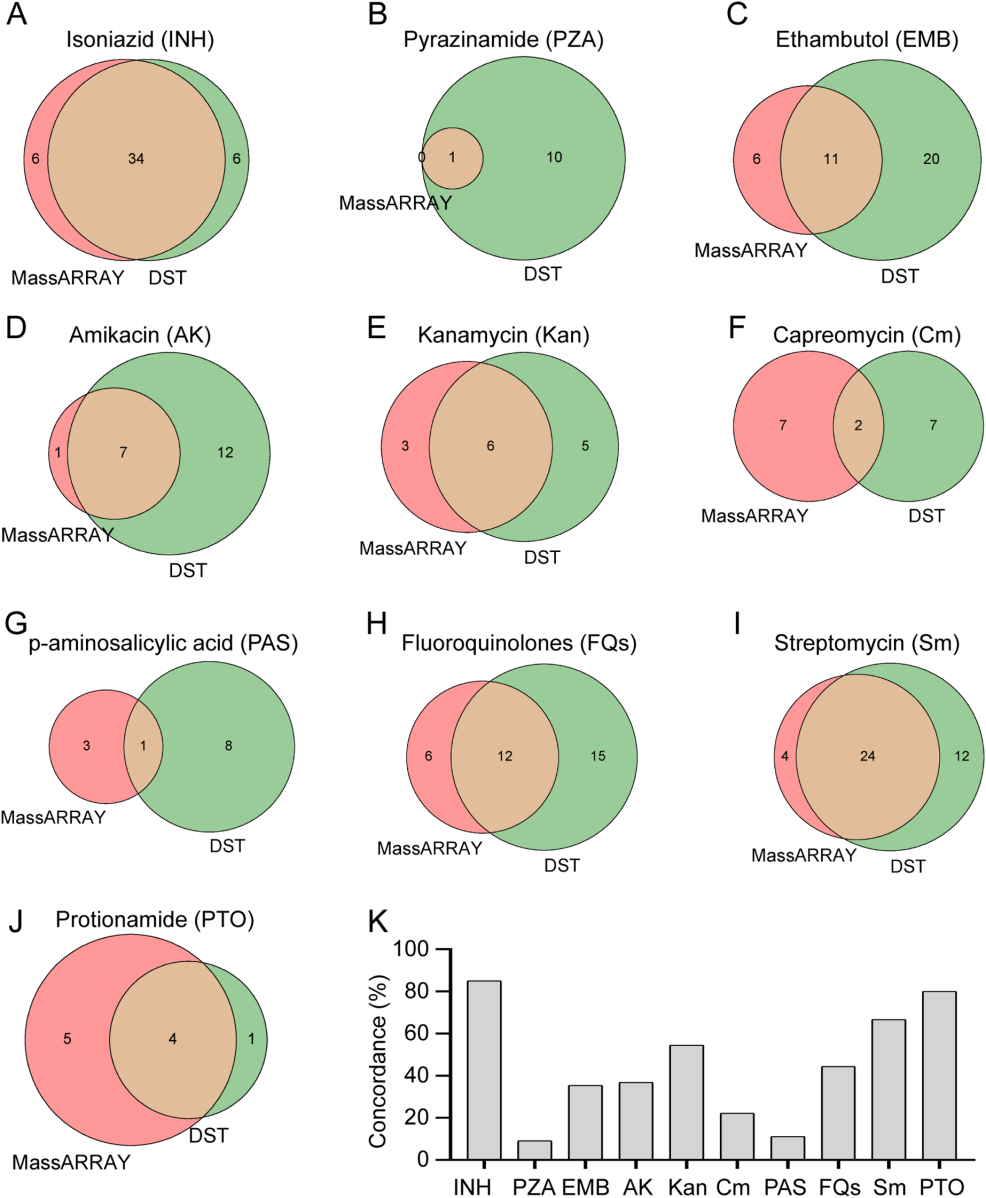
Comparative analysis of drug resistance detection methods. **(A-J)** Comparative performance of MassARRAY and Drug Susceptibility Testing (DST) methods for detection of resistance to various drugs: **(A)** Isoniazid, **(B)** Pyrazinamide, **(C)** Ethambutol, **(D)** Amikacin, **(E)** Kanamycin, **(F)** Capreomycin, **(G)** p-aminosalicylic acid, **(H)** Fluoroquinolones, **(I)** Streptomycin, **(J)** Protionamide. **(K)** Concordance analysis between MassARRAY and DST methods for detection of drug resistance in *M. tuberculosis*.

In addition to examining rifampicin, we also evaluated the efficacy of the MassARRAY technique in identifying drug resistance in *M. tuberculosis* towards alternative medications. We evaluated the efficacy of the MassARRAY approach with drug susceptibility testing (DST) in identifying drug resistance of *M. tuberculosis* to other medicines in 92 cases that were submitted to DST. The effectiveness of these two approaches in determining *M. tuberculosis* resistance to different anti-tuberculosis medications (such as isoniazid, pyrazinamide, ethambutol, amikacin, kanamycin, etc.) is depicted in the Venn diagram (**Figures 4A–J**). When it came to identifying *M. tuberculosis* resistance to isoniazid, kanamycin, streptomycin, and protionamide, the MassARRAY approach demonstrated strong concordance when compared to DST, with percentages of 85%, 54.5%, 66.7%, and 80%, respectively. However, concordance was lower, at 9.1%, 22.2%, and 11.1%, for detecting *M. tuberculosis* resistance to pyrazinamide, capreomycin, and p-aminosalicylic acid, respectively (**Figure 4K**). Additionally, DST found that, of the 92 patients, there were 7, 3, and 2 cases of *M. tuberculosis* resistance to linezolid, cycloserine, and clofazimine, respectively, whereas the MassARRAY approach was unable to identify resistance to any of these three medications (**Supplementary Table S1**). According to our findings, the MassARRAY approach discloses flaws in some anti-tuberculosis medications while demonstrating excellent accuracy and dependability in others.

### 3.4 Analysis of drug resistance genomic loci in *M. tuberculosis* detected by MassARRAY technology

**Figure 5:**
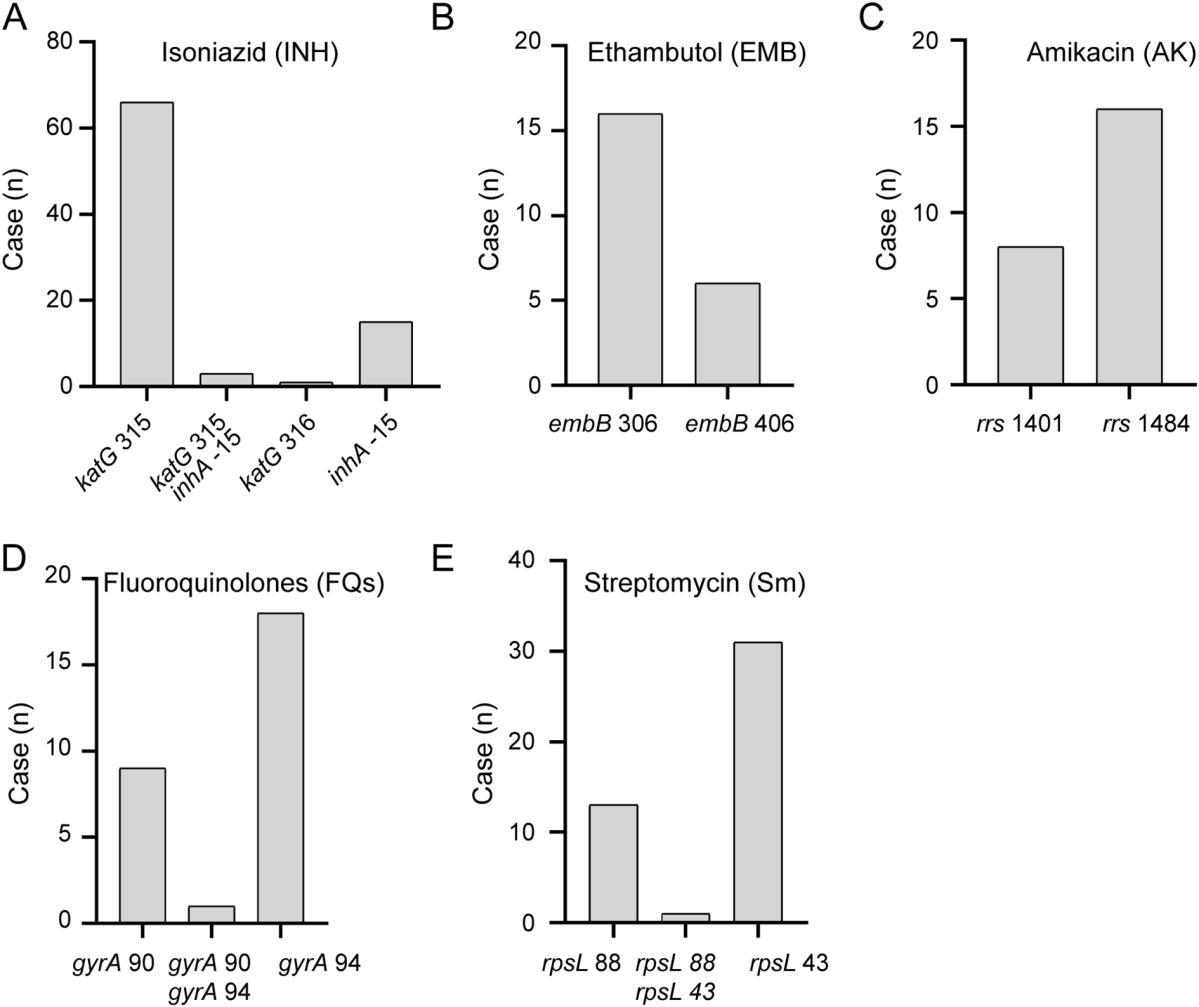
Distribution of Resistance Genomic Loci Detected by MassARRAY. Distribution of resistance genomic loci detected by MassARRAY for various drugs: **(A)** Isoniazid, **(B)** Ethambutol, **(C)** Amikacin, **(D)** Fleoroquinolones, **(E)** Streptomycin.

Utilizing 289 patient samples that underwent MassARRAY analysis, we analyzed the distribution of drug-resistant genetic mutation sites in *M. tuberculosis* and performed a comprehensive analysis. From the 289 patient samples that underwent MassARRAY analysis, resistance to isoniazid, ethambutol, fluoroquinolones, streptomycin, and amikacin was detected in 85, 22, 28, 45, and 24 cases, respectively (**Supplementary Table S1**). Notably, the mutation at locus *katG*315 was the most common among isoniazid-resistant variants, making up 81.1% of the total (**Figure 5A**). The most frequent mutation among ethambutol-resistant strains was found at locus *embB*306, accounting for 72.7% of the total alterations (**Figure 5B**). The most common mutation loci for resistance to amikacin, streptomycin, and fluoroquinolones were *rrs*1484, *rpsL*43, and *gyrA*94, respectively (**Figures 5C–E**). These findings underline the immense potential of MassARRAY even further. These results further highlight the significant potential of MassARRAY technology in identifying critical genetic loci associated with drug resistance in *M. tuberculosis*.

## 4 Discussion

The effectiveness of MassARRAY in identifying *M. tuberculosis* and detecting its drug resistance was evaluated in this study. Our findings show that both MassARRAY and Xpert exhibit high sensitivity (**Figure 2B**), confirming their effectiveness as diagnostic tools for detecting true positive cases of MTB. Additionally, the high negative predictive values (NPV) suggest that these methods are reliable in excluding MTB infection in patients when results are negative. These results align with previous studies (Akyar et al., 2018; Zhao et al., 2021; Wang et al., 2023), supporting the robustness of the methods. The key advantage of MassARRAY stems from the integration of MALDI-TOF MS with multiplex PCR amplification, which facilitates high-throughput, rapid, and precise detection of specific MTB nucleic acid sequences (Robinne et al., 2022).

However, the specificities of both MassARRAY and Xpert are relatively low (**Figure 2B**). This may be due to several factors, first, false positives might arise from contamination during sample preparation or cross-reactivity with non-tuberculous mycobacteria (NTM). Second, the use of culture as the gold standard has limitations, particularly in patients with low bacterial loads or prior antibiotic exposure, which may underestimate true MTB positivity. Notably, many patients in our study were referred to our hospital after receiving initial treatment at smaller local hospitals, where they commonly received antibiotics, including fluoroquinolones and other anti-tuberculosis drugs. These treatments likely reduced their bacterial load, making it more difficult to culture *M. tuberculosis* from their samples. To mitigate these issues, we implemented strict laboratory protocols, including separate handling of samples for different diagnostic methods. Additionally, MassARRAY incorporates propidium monoazide (PMA), which binds to DNA in non-viable cells and prevents its amplification during PCR, thereby reducing false positives from contamination or non-viable bacteria (TruChado et al., 2016).

We also investigated the application of MassARRAY in detecting drug resistance in MTB. The findings demonstrate that MassARRAY has a high level of accuracy in identifying resistance to anti-tuberculosis medications. Specifically, the resistance rates for rifampicin, isoniazid, and streptomycin were 34.3%, 39.4%, and 22.2%, respectively, among the 216 MTB-positive cases detected by MassARRAY. In comparison, resistance rates for these drugs were 40.0%, 41.2%, and 37.6% among the 85 MTB-positive cases detected through culture. Xpert detected rifampicin resistance in 35.9% of the 156 MTB-positive cases (**Supplementary Table S1**). This result aligns with the study by Liang et al., which showed that MassARRAY is as sensitive and specific as Xpert MTB/RIF at identifying rifampicin resistance (Liang et al., 2024). Additionally, our findings were compared to a 2021 epidemiological study that analyzed drug resistance in Henan Province across 30 counties. This study reported the highest resistance rates among retreatment patients for streptomycin (23.5%), isoniazid (25.14%), rifampicin (19.67%), ofloxacin (14.75%), ethambutol (14.21%), moxifloxacin (12.02%), levofloxacin (10.93%), kanamycin (2.73%), and amikacin (2.73%) (Zhu Yankun et al., 2024). Both our study and the epidemiological report highlight rifampicin, isoniazid, and streptomycin as having the highest resistance rates, suggesting that these are the predominant resistant strains circulating in Henan Province.

We identified several key genetic loci associated with drug resistance, such as *katG315* for isoniazid and *rpoB531* for rifampicin, which has significant clinical implications. These loci enable the development of personalized treatment regimens by predicting resistance patterns and guiding the selection of effective drug combinations. For example, detecting *katG315* mutations may prompt clinicians to avoid isoniazid in favor of alternative drugs, thereby improving treatment efficacy. Furthermore, analysis of co-occurring mutations, such as rpoB 531 with katG 315, could provide insights into multi-drug resistance patterns, which are critical for optimizing treatment strategies in MDR-TB cases. Future studies should explore these patterns to enhance the clinical relevance of molecular diagnostics, thereby highlighting the potential of incorporating MassARRAY into routine clinical workflows to advance precision medicine for TB management. However, our results also underscored the limitations of MassARRAY in detecting resistance to linezolid, cycloserine, and clofazimine, which could stem from unknown or less characterized resistance loci not currently covered by the assay (Oviaño and Bou, 2018). Future studies should focus on identifying new resistance loci for second-line drugs, such as pyrazinamide and clofazimine, and incorporating these loci into the MassARRAY platform to expand its detection capabilities. Ongoing research, including efforts aligned with the WHO’s 2023 ‘Catalog of Mutations Associated with Drug Resistance in *Mycobacterium tuberculosis* (WHO, 2023b),’ could further enhance the assay’s ability to detect resistance to emerging anti-tuberculosis drugs, thereby broadening its clinical utility.

For the purpose of directing precise clinical pharmaceutical use, improving treatment success rates, and lowering the risk of disease transmission, rapid and reliable drug resistance testing is essential (Huang et al., 2013; Martinez et al., 2014). Quick access to comprehensive resistance data is provided by MassARRAY, which helps physicians develop individualized treatment plans that improve patient outcomes and slow the spread of resistant strains (Ioerger et al., 2009; Van Belkum and Dunne, 2013). Despite the robust findings, the sample size (289 cases) limits the generalizability of the results. A larger sample size would enhance the precision of these estimates and improve the statistical power of comparisons between methods. To validate the broader applicability of MassARRAY, future studies will include multicenter trials across diverse geographical regions, including low-resource settings with higher prevalence of MDR-TB and XDR-TB. These trials will evaluate the platform’s performance in various demographic and clinical contexts, ensuring generalizability and scalability. Additionally, the cost-effectiveness of MassARRAY will be assessed in such settings to establish its feasibility for large-scale implementation. Further studies could improve the precision and reliability of the MassARRAY by expanding the sample size, conducting multicenter collaborations, refining the testing processes, and developing new sites for resistance detection (Mugenyi et al., 2024), this would help verify its clinical value in tuberculosis across various populations.

In conclusion, this study show that MassARRAY has the potential to be a quick, precise, high-throughput technique for identifying *M. tuberculosis* and detecting drug resistance to several first- and second-line anti-tuberculosis medications. MassARRAY could significantly enhance TB diagnostic and management techniques, especially in resource-limited settings. However, further studies are needed to confirm its efficacy in various populations and environments and investigate its potential for identifying medication resistance to newer anti-tuberculosis drugs. Addressing these research gaps will enable the full utilization of MassARRAY in the global fight against tuberculosis and ultimately improve patient outcomes.

## Data Availability

The original contributions presented in the study are included in the article/**Supplementary Material**. Further inquiries can be directed to the corresponding authors.

## References

[B1] Abe C. Hirano K. Tomiyama T. (1999). Simple and rapid identification of the Mycobacterium tuberculosis complex by immunochromatographic assay using anti-MPB64 monoclonal antibodies. J. Clin. Microbiol. 37, 3693–3697. doi: 10.1128/JCM.37.11.3693-3697.1999, PMID: 10523576 PMC85727

[B2] Akyar I. Çavuşoğlu C. Ayaş M. Sürücüoğlu S. İlki Z. A. Kaya D. E.. (2018). Evaluation of the performance of MALDI-TOF MS and DNA sequence analysis in the identification of mycobacteria species. Turkish J. Med. Sci. 48, 1351–1357. doi: 10.3906/sag-1801-198, PMID: 30543090

[B3] Aljohani A. S. M. (2023). Botanical compounds: A promising approach to control mycobacterium species of veterinary and zoonotic importance. Pakistan Veterinary J. 43 (6), 633–642. doi: 10.29261/pakvetj/2023.107

[B4] Devonshire A. S. Honeyborne I. Gutteridge A. Whale A. S. Nixon G. Wilson P.. (2015). Highly reproducible absolute quantification of Mycobacterium tuberculosis complex by digital PCR. Analytical Chem. 87, 3706–3713. doi: 10.1021/ac5041617, PMID: 25646934

[B5] Dheda K. Gumbo T. Maartens G. Dooley K. E. McNerney R. Murray M.. (2017). The epidemiology, pathogenesis, transmission, diagnosis, and management of multidrug-resistant, extensively drug-resistant, and incurable tuberculosis. Lancet Respir. Med. doi: 10.1016/S2213-2600(17)30079-6, PMID: 28344011

[B6] Dong B. He Z. Li Y. Xu X. Wang C. Zeng J. (2022). Improved conventional and new approaches in the diagnosis of tuberculosis. Front. Microbiol. 13, 924410. doi: 10.3389/fmicb.2022.924410, PMID: 35711765 PMC9195135

[B7] Gao X. Li T. Han W. Xiong Y. Xu S. Ma H.. (2024). The positivity rates and drug resistance patterns of Mycobacterium tuberculosis using nucleotide MALDI-TOF MS assay among suspected tuberculosis patients in Shandong, China: a multi-center prospective study. Front. Public Health 12, 1322426. doi: 10.3389/fpubh.2024.1322426, PMID: 38304182 PMC10830759

[B8] Hanna B. A. Ebrahimzadeh A. Elliott L. B. Morgan M. A. Novak S. M. Rusch-Gerdes S.. (1999). Multicenter evaluation of the BACTEC MGIT 960 system for recovery of mycobacteria. J. Clin. Microbiol. 37, 748–752. doi: 10.1128/JCM.37.3.748-752.1999, PMID: 9986844 PMC84542

[B9] Huang A. M. Newton D. Kunapuli A. Gandhi T. N. Washer L. L. Isip J.. (2013). Impact of rapid organism identification via matrix-assisted laser desorption/ionization time-of-flight combined with antimicrobial stewardship team intervention in adult patients with bacteremia and candidemia. Clin. Infect. Dis. 57, 1237–1245. doi: 10.1093/cid/cit498, PMID: 23899684

[B10] Ioerger T. R. Koo S. No E.-G. Chen X. Larsen M. H. Jacobs W. R. Jr.. (2009). Genome analysis of multi-and extensively-drug-resistant tuberculosis from KwaZulu-Natal, South Africa. PloS One 4, e7778. doi: 10.1371/journal.pone.0007778, PMID: 19890396 PMC2767505

[B11] Irfan M. Bisht D. (2022). Innovations in molecular identification of mycobacterium tuberculosis. J. Pure Appl. Microbiol. doi: 10.22207/JPAM.16.1.76

[B12] Li B. Zhu C. Sun L. Dong H. Sun Y. Cao S.. (2022). Performance evaluation and clinical validation of optimized nucleotide MALDI-TOF-MS for mycobacterial identification. Front. Cell. Infect. Microbiol. 12, 1079184. doi: 10.3389/fcimb.2022.1079184, PMID: 36530426 PMC9755490

[B13] Liang R. Li J. Zhao Y. Qi H. Bao S. Wang F.. (2024). A comparative study of MassARRAY and GeneXpert assay in detecting rifampicin resistance in tuberculosis patients’ clinical specimens. Front. Microbiol. 15, 1287806. doi: 10.3389/fmicb.2024.1287806, PMID: 38384275 PMC10879633

[B14] Martinez R. M. Bauerle E. R. Fang F. C. Butler-Wu S. M. (2014). Evaluation of three rapid diagnostic methods for direct identification of microorganisms in positive blood cultures. J. Clin. Microbiol. 52, 2521–2529. doi: 10.1128/JCM.00529-14, PMID: 24808235 PMC4097746

[B15] Mechal Y. Benaissa E. El Mrimar N. Benlahlou Y. Bssaibis F. Zegmout A.. (2019). Evaluation of GeneXpert MTB/RIF system performances in the diagnosis of extrapulmonary tuberculosis. BMC Infect. Dis. 19, 1–8. doi: 10.1186/s12879-019-4687-7, PMID: 31856744 PMC6924055

[B16] Mugenyi N. Ssewante N. Baruch Baluku J. Bongomin F. MuKenya Irene M. Andama A.. (2024). Innovative laboratory methods for improved tuberculosis diagnosis and drug-susceptibility testing. Front. Tuberculosis 1, 1295979. doi: 10.3389/ftubr.2023.1295979 PMC13494940 42638753

[B17] Oviaño M. Bou G. (2018). Matrix-assisted laser desorption ionization–time of flight mass spectrometry for the rapid detection of antimicrobial resistance mechanisms and beyond. Clin. Microbiol. Rev. 32(1), e00037–18. doi: 10.1128/CMR.00037-18, PMID: 30487165 PMC6302359

[B18] Robinne S. Saad J. Morsli M. Hamidou Z. H. Tazerart F. Drancourt M.. (2022). Rapid identification of Mycobacterium tuberculosis complex using mass spectrometry: a proof of concept. Front. Microbiol. 13, 753969. doi: 10.3389/fmicb.2022.753969, PMID: 35432257 PMC9008353

[B19] Shi J. He G. Ning H. Wu L. Wu Z. Ye X.. (2022). Application of matrix-assisted laser desorption ionization time-of-flight mass spectrometry (MALDI-TOF MS) in the detection of drug resistance of Mycobacterium tuberculosis in re-treated patients. Tuberculosis 135, 102209. doi: 10.1016/j.tube.2022.102209, PMID: 35550524

[B20] Sun X. Song J. Leng X. Li F. Wang H. He J.. (2023). A preliminary evaluation of targeted nanopore sequencing technology for the detection of Mycobacterium tuberculosis in bronchoalveolar lavage fluid specimens. Front. Cell. Infect. Microbiol. 13. doi: 10.3389/fcimb.2023.1107990, PMID: 38029234 PMC10668825

[B21] TruChado P. Gil M. I. Kostic T. Allende A. (2016). Optimization and validation of a PMA qPCR method for Escherichia coli quantification in primary production. Food Control 62, 150–156. doi: 10.1016/j.foodcont.2015.10.014

[B22] Van Belkum A. Dunne W. M. Jr (2013). Next-generation antimicrobial susceptibility testing. J. Clin. Microbiol. 51, 2018–2024. doi: 10.1128/JCM.00313-13, PMID: 23486706 PMC3697721

[B23] Vogelstein B. Kinzler K. W. (1999). Digital pcr. Proc. Natl. Acad. Sci. 96, 9236–9241. doi: 10.1073/pnas.96.16.9236, PMID: 10430926 PMC17763

[B24] Wang Y. Xu Q. Xu B. Lin Y. Yang X. Tong J.. (2023). Clinical performance of nucleotide MALDI-TOF-MS in the rapid diagnosis of pulmonary tuberculosis and drug resistance. Tuberculosis, 143, 102411. doi: 10.1016/j.tube.2023.102411, PMID: 37748279

[B25] WHO (2020). WHO consolidated guidelines on tuberculosis. Module 3: Diagnosis-Rapid diagnostics for tuberculosis detection (Geneva: World Health Organization.). 33999549

[B26] WHO (2023a). Global tuberculosis report 2023 (Geneva: World Health Organization).

[B27] WHO (2023b). Catalogue of mutations in Mycobacterium tuberculosis complex and their association with drug resistance (Geneva: World Health Organization).

[B28] Wu X. Tan G. Yang J. Guo Y. Huang C. Sha W.. (2022). Prediction of Mycobacterium tuberculosis drug resistance by nucleotide MALDI-TOF-MS. Int. J. Infect. Dis. 121, 47–54. doi: 10.1016/j.ijid.2022.04.061, PMID: 35523300

[B29] Yang H. Li A. Dang L. Kang T. Ren F. Ma J.. (2023). A rapid, accurate, and low-cost method for detecting Mycobacterium tuberculosis and its drug-resistant genes in pulmonary tuberculosis: applications of MassARRAY DNA mass spectrometry. Front. Microbiol. 14, 1093745. doi: 10.3389/fmicb.2023.1093745, PMID: 36910195 PMC9996023

[B30] Yao L. Gui X. Wu X. Yang J. Fang Y. Sun Q.. (2023). Rapid Identification of nontuberculous mycobacterium species from respiratory specimens using nucleotide MALDI-TOF MS. Microorganisms 11, 1975. doi: 10.3390/microorganisms11081975, PMID: 37630537 PMC10458091

[B31] Yoon E.-J. Jeong S. H. (2021). MALDI-TOF mass spectrometry technology as a tool for the rapid diagnosis of antimicrobial resistance in bacteria. Antibiotics 10, 982. doi: 10.3390/antibiotics10080982, PMID: 34439032 PMC8388893

[B32] Zhao H. Yang Y. Lyu J. Ren X. Cheng W. (2021). Development and application of a method to detect 27 respiratory pathogens using multiplex RT-PCR combined with MassARRAY technology. BMC Infect. Dis. 21, 1–8. doi: 10.1186/s12879-021-06404-0, PMID: 34433411 PMC8385475

[B33] Zhu Yankun S. R. Chang W. Zhang Y. Ma X. Zheng D. Wang S.. (2024). Drug resistance characteristics of 1–277 isolates of Mycobacterium tuberculosis in Henan province in 2021. Modern Dis. Control Prev. 35, 1–5.

